# A Comparison of the Resistance- and Capacitance-Based Sensing of Geopolymer and Cement Composites with Graphite Filler Under Compression

**DOI:** 10.3390/ma18040750

**Published:** 2025-02-08

**Authors:** Pavel Rovnaník, Ivo Kusák, Pavel Schmid, Patrik Bayer

**Affiliations:** 1 Institute of Chemistry, Faculty of Civil Engineering, Brno University of Technology, 602 00 Brno, Czech Republic; patrik.bayer@vut.cz; 2 Institute of Physics, Faculty of Civil Engineering, Brno University of Technology, 602 00 Brno, Czech Republic; ivo.kusak@vut.cz; 3 Institute of Building Testing, Faculty of Civil Engineering, Brno University of Technology, 602 00 Brno, Czech Republic; pavel.schmid@vut.cz

**Keywords:** slag, fly ash, Portland cement, graphite, self sensing, capacitance, resistance, mechanical properties, microstructure

## Abstract

Aluminosilicate binders, such as Portland cement or geopolymers, are generally considered electrical insulators. In order to decrease their electrical resistance, electrically conductive fillers are added. This brings new application possibilities, such as the self-sensing and self-monitoring of smart structures. In this study, three different aluminosilicate composites with the same amount of fine graphite filler (6% with respect to the basic aluminosilicate raw material) were tested for resistance- and capacitance-based self-sensing properties. Portland cement and two geopolymer binders were used as the basic matrices for the conductive composites. The composites were tested for self-sensing properties in repeated compression in the elastic area, static mechanical properties, and microstructure using scanning electron microscopy and mercury intrusion porosimetry. The results showed that alkali-activated materials are less stiff than Portland cement composite; however, they provide better self-sensing properties, regardless of the measured electrical parameters. The highest capacitance-based gauge factor 74.5 was achieved with the blended slag/fly ash geopolymer composite, whereas the cement composite showed very poor sensitivity, with a gauge factor of 10.2. The study showed a new possibility of self-sensing based on the measurement of capacitance, which is suitable for geopolymers and alkali-activated composites; however, in the case of cement composites, it is very limited.

## 1. Introduction

The development of smart materials has gained significant attention in recent years, particularly in civil engineering. Among these, self-sensing materials, which can monitor their own structural health, have shown several key effects on civil engineering and infrastructure development [1]. First, self-sensing materials can continuously monitor the structural integrity of buildings, bridges, and other infrastructures. These real-time data aid in the early detection of damage, such as cracks or stress points, allowing for timely maintenance and repair [2,3,4]. By providing immediate feedback on structural health, these materials can also prevent catastrophic failures and enhance the overall safety of structures. The early detection of structural issues can significantly reduce maintenance costs by addressing problems before they become severe. This proactive approach minimizes the need for extensive repairs or replacements and leads to long-term cost savings [5].

Alkali-activated materials and geopolymers are alternatives to commonly used materials based on Portland cement. Since these materials can be prepared using either waste materials such as slag or fly ash, or natural pozzolanas, they reduce their environmental impact associated with CO_2_ emissions [6,7]. Alkali-activated materials provide superior durability compared to ordinary concrete in many aspects, such as a higher resistance to chloride penetration [8,9,10], chemical attack [11,12,13,14,15], freezing and thawing [16,17,18], and high temperatures [19,20,21]. It is expected that this concrete will be a versatile and effective material for use in a variety of construction projects, such as wastewater treatment plants, bridges, high-rise buildings, highways, tunnels, dams, hydraulic structures, and tunnel segments [22].

Aluminosilicate building materials, such as cementitious or geopolymer concrete, are generally considered electrical insulators. This characteristic is primarily due to their composition, which includes non-conductive components such as C-S-H in cementitious composites or C-A-S-H gels and amorphous zeolite-like structures in geopolymers. The conductivity of these materials is caused by soluble components that can dissociate in the presence of moisture to form ions that contribute to the ionic conductivity—Ca(OH)_2_ in cement-based materials and excessive non-bound alkalis in geopolymers. However, the conductivities of alkali-activated materials and geopolymers are much higher than those of cement-based materials. This is caused by several factors, among which the most notable are the concentration and ionic conductivity of dissolved ions and the pore structure with a higher portion of gel pores <10 nm, which contain condensed water even under very dry conditions [23,24].

The incorporation of conductive fillers, such as carbon nanotubes (CNTs), graphite, carbon black (CB), and carbon (CFs) or metallic fibres, into these matrices enhances their electrical properties and can impart self-sensing capabilities. These fillers create a conductive network within the composite, enabling it to detect and respond to mechanical stress, strain, and damage through changes in electrical parameters [25,26]. The critical parameters for the superior influence of the conductive fillers on the electrical and self-sensing properties are their concentration and uniform distribution within the composite material [27]. The electrical percolation threshold, an amount of conductive fillers beyond which the electrical resistance of cementitious composites drastically decreases, plays a significant role in self-sensing capability [28]. However, the type of the conductive admixture also affects the conductivity and self-sensing properties of the cementitious materials. Yoo et al. [29] showed that CFs provide better conductivity than CNTs at the same volume fraction content due to the broad conductive pathways but CNTs showed improved self-sensing ability under cyclic compression. Even better sensing performance was achieved with combined CF–CNT- or CF–graphite-modified composites [30,31].

The detection of self-sensing properties is typically based on piezoresistivity. When the composite is subjected to a compressive load, in the initial elastic region, the electrical resistance decreases as the conductive paths are deformed and increases again after the stress is released [25,32]. However, the resistivity is also affected by the temperature [33,34], internal moisture [35,36], and damage [2,3,37]. The elastic strain reversibly affects the resistivity, whereas damage irreversibly increases the resistivity. The irreversible resistivity change correlates with irreversible strain; thus, the damage effects can be distinguished. In cyclic loading at the same stress amplitude, each loading cycle may cause minor damage, resulting in an irreversible increase in resistivity [38]. This indicates a high level of effectiveness in detecting even minor damage.

When the temperature increases, the resistivity decreases due to the activation energy required for electrons to jump across the interfaces in cementitious materials. In composites with conductive fillers, the relevant interfaces are the filler–matrix and filler–filler interfaces. The temperature effect causes these materials to function as thermistors.

The internal moisture of cementitious composites contributes to the ionic conductivity of the matrix by the dissolution and dissociation of soluble species. When the moisture content increases, the resistivity decreases. This function is not linear and also depends on the conductive filler dosage [35].

All three factors—elastic strain, moisture, and temperature—have reversible effects on resistivity. As a result, their resolution requires additional means, such as additional means for measuring temperature and humidity.

The detection of self-sensing properties based on the measurement of capacitance was first reported by Fu et al. [39]. The applied compressive strain was reflected in an increase in the electrical capacitance when the electrodes in the sandwich configuration were used. Ozturk [40] reported that this type of sensing was superior to resistance-based self-sensing. The second type of self-sensing uses a coplanar configuration of electrodes attached to the surface of the material, and the capacitance is measured in the transverse direction. Axial compression is associated with transverse tension owing to Poisson’s effect, and the capacitance decreases [41,42,43]. Chung and Wang [44] also showed that this type of capacitance-based self-sensing can be performed on a material that does not require any conductive admixture and proved the excellent sensitivity of this type of self-sensing.

Another technique for assessing self-sensing properties is impedance spectroscopy. AC impedance measurements are more accurate and reliable than DC resistance measurements because of the elimination of the polarization effect and negligible electrode contact resistance. AC systems can be used for impedance measurements, which allow for resistance, capacitance, and inductance characterization, whereas DC systems can only be used for resistance measurements [45,46,47].

Although most of the research has focused on cement-based composites, several research papers have also been recently devoted to alkali-activated materials and geopolymers [48,49,50,51,52,53,54]. Luo et al. [55] investigated the self-sensing properties of cement and alkali-activated fly ash/slag based composite with 15% of graphite powder and found a lower sensitivity compared to the plain material without graphite but they pointed out that attention should be paid to the conductive filler amount. Sufficient self-sensing ability in compression was also achieved for alkali-activated fly ash/slag composites with a small amount of steel fibres (<0.8%) [56]. Ma et al. [57] recently showed that even the type aluminosilicate matrix has considerable influence on the electrical resistivity and self-sensing properties of composites with CFs. While the addition of metakaolin to the slag geopolymer improved the conductivity, the piezoresistivity was poor. On the other hand, the addition of fly ash or silica fume had a positive effect on piezoresistivity. However, capacitance-based self-sensing of alkali-activated materials or geopolymers has not been reported yet. The primary objective of this study was to investigate the effectiveness of various electrical parameters obtained by the AC impedance-based technique in determining the self-sensing properties of aluminosilicate building composites with graphite filler as a conductive admixture. Two types of geopolymers based on ground-granulated blast furnace slag and fly ash from the high-temperature combustion of black coal were compared with a composite with a Portland cement matrix. The amount of graphite conductive filler was adjusted so that it was below the percolation threshold, which is favourable for the self-sensing performance. This study provides new insights into the ability of different electrical parameters to detect the mechanical load and structural changes in alkali-activated materials.

## 2. Materials and Methods

### 2.1. Materials and Mixing Procedure

Three types of basic aluminosilicate precursors were used in this study: Portland cement CEM I 42.5 R (Heidelberg Cement, Mokrá, Czech Republic), ground granulated blast furnace slag (Kotouč a.s., Štramberk, Czech Republic), and fly ash from the high-temperature combustion of black coal (Dětmarovice power plant, Dětmarovice, Czech Republic). The chemical composition of the aluminosilicate precursors is given in Table 1. Solid hydrous sodium disilicate Britesil C205 (PQ Corporation, Warrington, UK) was used as an alkaline activator to produce the slag and fly ash geopolymers. The composition of the activator comprises 53.40% SiO_2_ and 25.85% Na_2_O (SiO_2_/Na_2_O = 2.13). In order to enhance the electrical properties of the plain aluminosilicate materials, a PMM-11 graphite powder (Kooh-i-Noor, České Budějovice, Czech Republic) with a mean particle size of 8.1 (*d*_10_ = 3.1 μm, *d*_90_ = 17.6 μm) was used. Since graphite is hydrophobic, a 2% aqueous solution of Triton X-100 surfactant (Sigma-Aldrich, St. Louis, MO, USA) was applied for better dispersion of graphite particles. A 1% solution of silicone-based air detraining agent, Lukosan S (Lučební závody a.s., Kolín, Czech Republic), was added to reduce the foaming effect of Triton X-100. Standard quartz sand with a maximum grain size of 2 mm was used to prepare the mortars for testing their self-sensing and mechanical properties.

The composition of mixtures for the preparation of the test specimens is presented in Table 2. The graphite conductive filler dosage was adjusted to 6 wt.% with respect to the basic aluminosilicate raw material. This amount is below the percolation threshold, which is necessary to achieve self-sensing properties [54]. Fresh mixtures were prepared using a standard Hobart A-200 mixer (Hobart, Offenburg, Germany) and the following procedures were applied. First, the graphite powder was mixed with a part of water and Triton X-100 solution to ensure the proper dispersion of graphite. In the case of cement composite, Portland cement was added and the mixture was then stirred for 3 min. to prepare a homogenous paste. Subsequently, quartz sand, Lukosan S solution, and the rest of the mixing water were added, and the mixture was stirred for another 5 min. For the geopolymer mixtures, the activator was dissolved in another portion of the mixing water and mixed with the graphite suspension. Subsequently, an aluminosilicate precursor (slag, fly ash) was added, and the mixture was stirred for 3 min. to prepare a homogenous paste. Finally, quartz sand and Lukosan S solution were added and the slurry was stirred for another 5 min. The amount of mixing water was adjusted in order to have the same consistency for all mixtures. The basic composition of the mixtures and the amount of supporting admixtures were based on our previous study [54].

**Figure 1:**
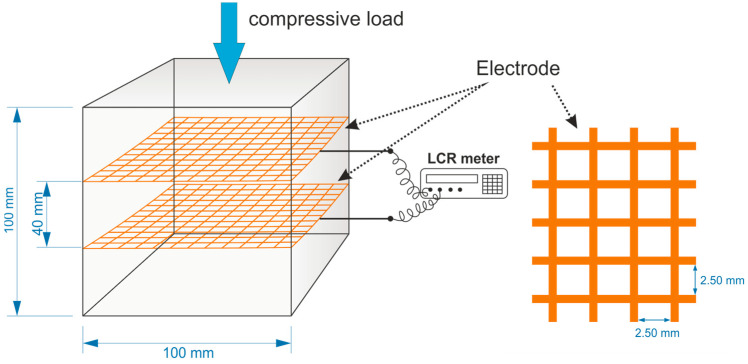
Experimental setup for self-sensing measurements during compressive loading tests [24].

The fresh slurries were cast into prismatic moulds (40 × 40 × 160 mm) for the determination of the mechanical properties and cubic moulds (100 × 100 × 100 mm) for the determination of the self-sensing properties. Two copper gauze electrodes were embedded in the samples to perform the electrical measurements (Figure 1). After 24 h, the hardened specimens were immersed in a water bath at 20 °C for 27 days. Before the measurement of the self-sensing properties, the cubic specimens were stored under ambient conditions for 30 days before testing to reach ambient humidity. 

### 2.2. Testing Methods

Prismatic specimens were used to determine mechanical properties. The flexural strengths were determined using a standard three-point bending test with a 100 mm span, and the compressive strengths were measured on the far edge of each of the two residual pieces obtained from the flexural test according to the EN 196-1 standard [58]. The results were obtained from three replicates.

The self-sensing properties were measured on cubic specimens using a LabTest^®^ 6.250 (Labortech, Opava, Czech Republic) precise electromechanical testing machine, which enables a maximum working load of 250 kN, and a GW INSTEK LCR-6000 Series multimeter (Good Will Instrument Co., Ltd., New Taipei, Taiwan) that can operate over a wide range of frequencies (10 Hz to 300 kHz) and test signal levels (10.00 mV to 2.00 V and 100.0 μA to 20.00 mA). The measurement was performed at a frequency of 1 kHz to eliminate the polarization of the electrodes. The samples were fitted with a strain gauge to measure the deformations in the direction parallel to the loading force and were loaded perpendicular to the plane of the Cu electrodes (Figure 1). In the experiment, the cyclic loading and releasing of the samples was performed linearly with a loading rate of 300 N·s^−1^ and in the range of 0.5–10 kN, which corresponds to the elastic part of the loading. The electrical resistance and capacitance values were recorded during loading. Each sample was subjected to 10 cycles.

Porosity was measured by means of mercury intrusion porosimetry analysis (MIP), which was conducted on the cut samples using a Micromeritics Poresizer 9310 porosimeter (Norcross, GA, USA). This device can generate a maximum pressure of 207 MPa and evaluate a theoretical pore diameter of 0.006 μm. The MIP test is performed in two steps: the low-pressure step first evacuates gasses, fills the sample holder with mercury, and performs porosimetry from about 7 to 179 kPa; the high-pressure step reaches pressures between 414 kPa and 207 MPa. The contact angle and surface tension assumed for all tests were 130° and 485 mN·m^−1^, respectively.

The morphology of the composites was analyzed on the fracture surface using a TESCAN MIRA3 XMU scanning electron microscope (Brno, Czech Republic) in SE mode. The experiments were carried out on samples that were sputtered with gold. Samples for the MIP and SEM analyses were dried in an electric oven at 50 °C for 120 h prior to measurement in order to release free moisture.

## 3. Results

### 3.1. Mechanical Properties

**Figure 2:**
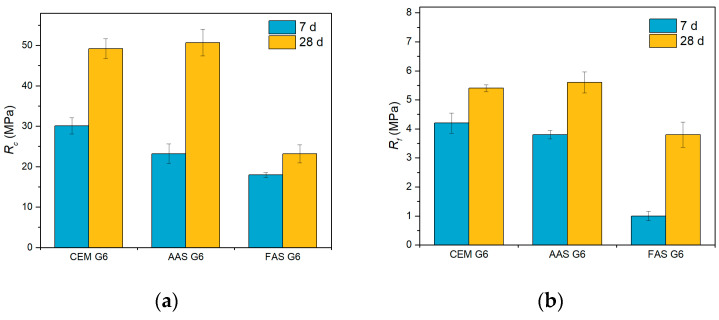
Mechanical properties of tested composites at the age of 7 and 28 days: (**a**) compressive strength; (**b**) flexural strength.

The mechanical properties of the tested composites were determined at the age of 7 and 28 days. The mechanical parameters were obtained for three specimens of each mixture, and the mean values, together with error bars representing the standard deviation, are presented in Figure 2. The highest 7-days compressive (30.1 MPa) and flexural (4.2 MPa) strengths were achieved for the cement-based composite. However, at the age of 28 days, a slightly better mechanical performance was achieved for the AAS composite. The compressive strength of this composite was 50.7 MPa and its flexural strength was 5.6 MPa. This implies that the reaction rate of the alkali-activated slag is slightly slower, but the final mechanical properties are comparable to those determined for cement-based composite. The blended fly ash/slag geopolymer composite showed the lowest values of both compressive and flexural strengths, which is in accordance with the lower reactivity of fly ash compared to blast furnace slag, although the dosage of the activator was doubled [59,60].

### 3.2. Mercury Intrusion Porosimetry

**Figure 3:**
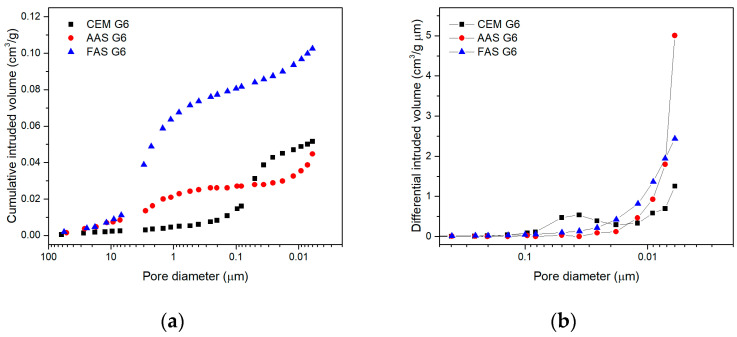
Distribution of pores determined by means of mercury intrusion porosimetry in range of pore diameters 0.006–60 μm: (**a**) cumulative intruded volume; (**b**) differential intruded volume.

The effect of the aluminosilicate precursor on the porosity of the tested composites was determined by mercury intrusion porosimetry in the pore diameter range of 0.006–60 μm. Figure 3 shows completely different pore distributions in all three mixtures. The highest total intruded porosity was observed for the fly ash/slag geopolymer composite. The pore volume of this material was more than twice that of other composites. This observation also explains its lower compressive strength because the volume of the capillary and large pores (>10 nm) has an adverse influence on the resistance to compression. Although the total intruded volumes of the AAS and CEM composites were similar, the pore distribution was very different. While cement composite had the majority of pore volume in the range of small- to medium-sized capillary pores (10–100 nm), large pores >1 μm and especially gel pores <10 nm dominated in the structure of AAS. This is also evident in the graph of the differential intruded volume, where the gel pores completely dominate the AAS structure.

### 3.3. Self-Sensing Properties

The self-sensing properties of structural materials are based on the change in the conductive network within the composites, so that the change in the electrical parameters can characterize the sensing behaviour. For these purposes, the fractional change in resistance (*FCR*) is usually used as a relative measure to describe the self-sensing properties under various loading conditions [1]. This can be calculated as follows:

(1) $$FCR(\%)=\frac{R-R_{0}}{R_{0}}\cdot 100$$

Similar to the electrical resistive response to an applied load, capacitance-based sensing was also tested in this study. The self-sensing properties are then described by the fractional change in capacitance (*FCC*) that can be calculated using the following equation:

(2) $$FCC(\%)=\frac{C-C_{0}}{C_{0}}\cdot 100$$

**Figure 4:**
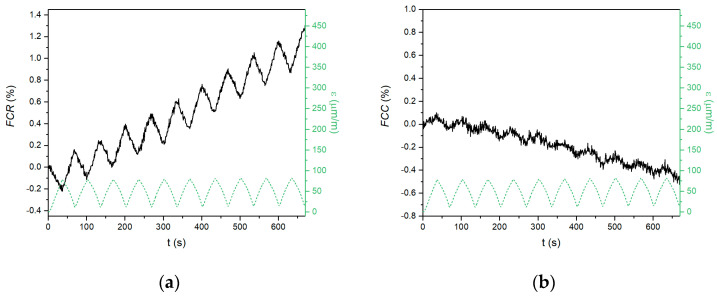
Self-sensing properties of CEM G6 composite during repeated compressive loading with constant amplitude recorded as (**a**) fractional change in resistance; (**b**) fractional change in capacitance.

**Figure 5:**
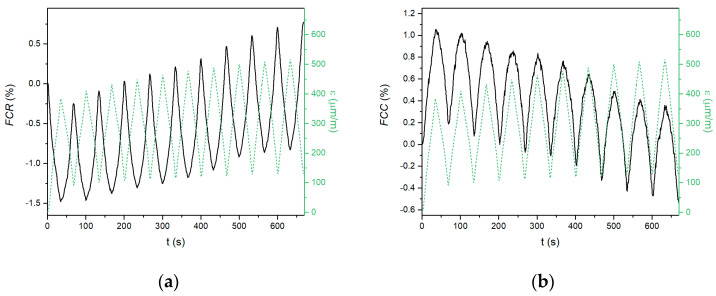
Self-sensing properties of AAS G6 composite during repeated compressive loading with constant amplitude recorded as (**a**) fractional change in resistance; (**b**) fractional change in capacitance.

**Figure 6:**
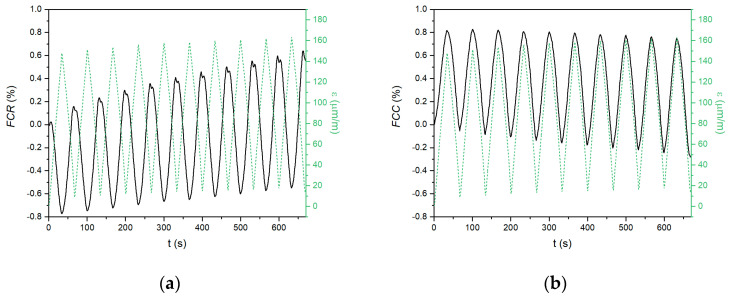
Self-sensing properties of FAS G6 composite during repeated compressive loading with constant amplitude recorded as (**a**) fractional change in resistance; (**b**) fractional change in capacitance.

When a compressive force is applied, the electrical properties of the conductive composite change. The changes in the electrical parameters and compressive strain during repeated loading of the tested materials are shown in Figure 4, Figure 5 and Figure 6. The left graphs represent resistance-based sensing, whereas the right graphs represent capacitance-based sensing. In the case of the Portland cement composite (Figure 4), the amplitude of the strain was low, reaching only 80 μm·m^−1^ at a 10 kN load, which indicates that the CEM G6 composite is quite stiff. When the compressive strain increased, the fractional change in the resistance decreased, and vice versa. The difference in resistance between the loaded and unloaded sample remained constant during repeated loading; however, the baseline of the curve, which is related to the initial resistance of the unloaded material, gradually increased. This phenomenon was observed in several previous works [26,38] and is associated with some permanent damage of the internal structure, although the permanent change in compressive strain was only 3.2 μm·m^−1^.

In the case of fractional change in capacitance, the quality of the signal was very low, with a very low signal/noise ratio, which is in contradiction to the observations reported by Ozturk [40]. However, some alterations in capacitance can be recognized. A gradual decrease in the initial capacitance during cyclic compressive loading has the same reason as that for the resistance-based measurement.

Composites based on the alkali-activated matrix, AAS G6 and FAS G6, showed similar behaviour; however, several essential differences were observed. First, the amplitudes of the compressive strain were much higher (386 μm·m^−1^ for AAS G6 and 148 μm·m^−1^ for FAS G6) than those for the CEM G6 composite, indicating a less stiff structure. For this reason, the electrical response to the applied load was also much larger. The amplitudes were up to 1.5% and 1.1% for resistance-based and capacitance-based sensing, respectively. Second, irreversible changes in the electrical parameters were observed for both alkali-activated composites, but the absolute difference was less pronounced than in the CEM G6 sample. The total increases in *FCR* after ten cycles were 1.3%, 0.8%, and 0.5% for CEM G6, AAS G6, and FAS G6, respectively. In the case of capacitance-based sensing, total decreases of 0.5, 0.5, and 0.3% were observed. For the alkali-activated composites, these permanent changes in the electrical parameters during compressive loading were also reflected by considerable permanent changes in compressive strain (124 μm·m^−1^ for AAS G6 and 14 μm·m^−1^ for FAS G6). However, the main difference lies in capacitance-based sensing. In contrast to cement-based composite, alkali-activated slag and fly ash/slag geopolymer composites exhibit excellent self-sensing response to applied load in capacitance measurements.

In order to express the sensitivity of the self-sensing properties to the strain response, a gauge factor (*GF*) can be used. It is usually defined as the fractional change in resistance per strain unit (3) and helps compare the self-sensing properties of different materials. Similarly, the fractional change in capacitance per strain unit (4) expresses the gauge factor for capacitance-based sensitivity.

(3) $${\mathrm{GF}}_{R}=\frac{∆R/R_{0}}{\varepsilon }$$

(4) $${\mathrm{GF}}_{C}=\frac{∆C/C_{0}}{\varepsilon }$$

**Figure 7:**
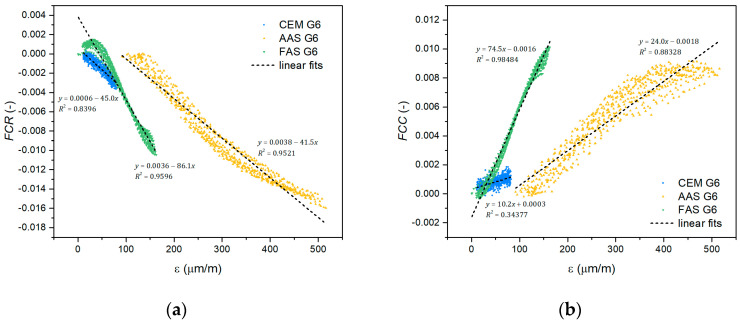
Electrical response of the tested composites vs. compressive strain recorded for (**a**) resistance-based sensing; (**b**) capacitance-based sensing. Linear fits for the self-sensing sensitivity dependence are depicted.

**Figure 8:**
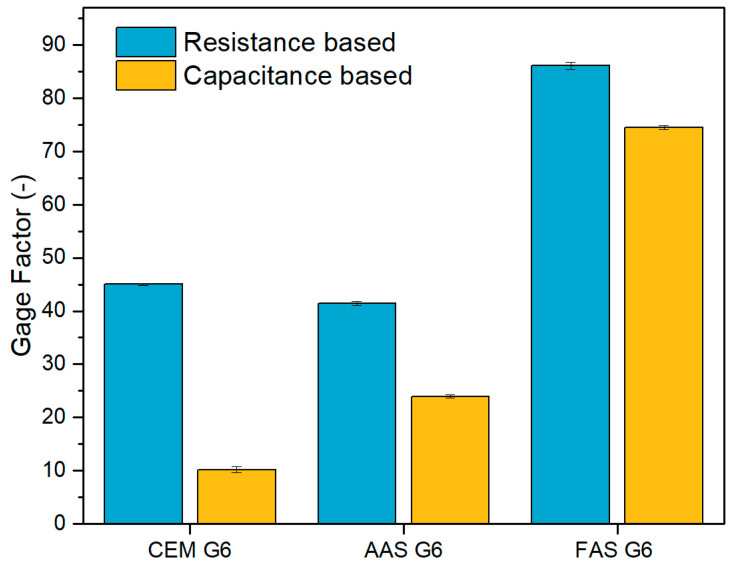
Calculated gage factors for resistance- and capacitance-based self-sensing properties.

The corresponding gauge factors were calculated as a slope from the linear fit curve of the dependence of *FCR* or *FCC* on strain (Figure 7). The error was evaluated using the standard deviation of the fit parameters. Baseline correction of the *FCR*, *FCC*, and strain data was performed to eliminate a gradual change in the measured parameters in the unloaded state during cyclic loading. The calculated gauge factors for the tested composites are presented in Table 3 and Figure 8. The results show that the resistance-based self-sensing ability is better than that based on the measurement of electrical capacitance. The resistance-based gauge factors of CEM G6 and AAS G6 composites are very similar, but the gauge factor of FAS G6 is more than twice as high. The capacitance-based gauge factor of CEM G6 was very low, indicating a very poor sensitivity of the capacitance change to the applied load, which is in accordance with the study published by Han et al. [61]. In the case of the AAS G6 composite, the capacitance-based sensitivity was approximately half the value achieved with the resistivity measurement. The best performance was achieved with the FAS G6 composite, where the gauge factor values were similar.

## 4. Discussion

Self-sensing properties of building materials are associated with alterations of their electrical properties when an external force is applied. This overall effect is a superposition of several factors that contribute to changes in the electrical parameters. When the composites are subjected to an external compressive force, the graphite filler is deformed, resulting in a change in its intrinsic resistance and capacitance. When the graphite particles are compressed, the distance between the carbon layer is reduced; hence, the width of tunnelling barrier decreases. The second factor stems from a change in the bonding between the functional filler and matrix or a change in the contact between the graphite particles, which results in a decrease in the contact resistance. Therefore, the electrical resistance decreases during compressive loading, and the fractional change in the resistance becomes negative. When the applied load is released, the material relaxes, and the initial resistance is regained.

Graphite particles dispersed in the aluminosilicate matrix also act as microcapacitators. There is plenty of microcapacitance due to the presence of ionic conduction caused by either calcium hydroxide solution in the cement matrix or free alkalis in alkali-activated materials. Aluminosilicate matrix is polarized when the electrical field is applied due to the accumulation of electrical charge on the surface of graphite particles. This process has already been described by Wang and Zhao [62], who investigated the self-sensing properties of cement composite with carbon fibres. Geopolymer and alkali-activated matrices generally exhibit higher basic capacitance than Portland cement owing to higher concentrations of mobile ionic particles [24]. When the composite is compressed, the distance between either the graphite particles or even single graphene layers in graphite decreases, which is followed by an increase in the capacitance according to the following equation:

(5) $$C=\varepsilon_{r}\cdot \varepsilon_{0}\frac{A}{d}$$

where *ε_r_* is the relative permittivity, *ε*_0_ is the permittivity constant, *d* is the distance between the graphite particles, and *A* is the area of the adjacent surfaces of the graphite particles. The capacitance decreases again when the particles move away from each other during unloading. This is quite different from capacitance-based measurement with coplanar electrodes that are attached to the surface and separated from the matrix by a dielectric film. In this case, the capacitance is in the plane of the material, and a normal compressive stress is followed by a decrease in the capacitance [41,63].

The essential difference in the sensitivity of capacitance-based sensing between the CEM G6 composite and the alkali-activated composites AAS G6 and, especially, FAS G6 lies in the considerable differences in the initial capacitance, which may arise from the differences in ionic conductivity. While the cement matrix contains poorly soluble portlandite, the alkali-activated matrix contains soluble and highly mobile alkali cations, which contribute to the higher ionic conductivity. The very low electrical resistance of the FAS G6 composite is caused by a higher dosage of the alkaline activator and may also be attributed to a higher level of internal moisture, which contributes to the dissolution and increase in the concentration of ionic species [35,64]. Moisture is also responsible for the increase in the initial capacitance [65,66].

**Figure 9:**
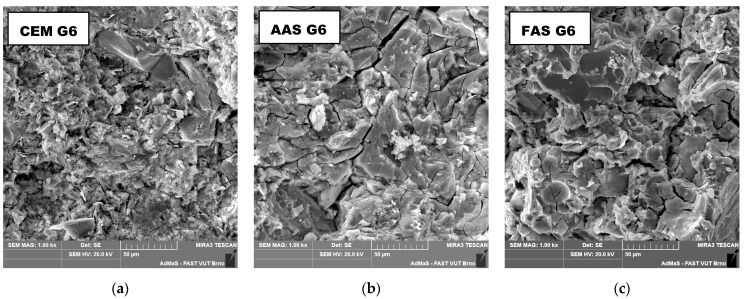
Morphology of tested composites depicted by SEM: (**a**) CEM G6; (**b**) AAS G6; (**c**) FAS G6. Microcracks are present in AAS and FAS matrices.

The increase in the baseline resistance and decrease in the baseline capacitance are associated with permanent structural changes in the aluminosilicate matrix. Alkali-activated composites seem to be more prone to permanent damage even at low compressive stress. This can be explained by the closing of microcracks perpendicular to the compressive stress, which commonly appear in the AAS matrix owing to their gel character and remarkable drying shrinkage [67,68]. These microcracks can be clearly recognized in the microstructure of both alkali-activated composites, whereas there are no obvious cracks in the cement matrix (Figure 9). This phenomenon is also reflected in the higher volume of larger pores (1–10 μm) determined by mercury intrusion porosimetry in AAS G6 and FAS G6, as previously described [24]. However, the applied stresses may result in the formation of additional longitudinal cracks due to Poisson’s effect, which causes permanent changes in electrical parameters. Although the permanent deformation of the cement composite was very low because the matrix is quite stiff and does not contain a large number of microcracks, the permanent change in the electrical parameters revealed changes in the microstructure. This observation shows that the analysis of changes in the electrical properties is able to detect changes in the microstructure or permanent damage, even though other mechanical parameters remain unchanged.

The porosimetry analysis showed significant differences in the total pore volume and the pore size distribution of tested composites, which affect both the early-age and long-term mechanical properties. In particular, a higher total porosity of slag/fly ash geopolymer composite may be related to a higher permeability of the material and action of aggressive environment. However, geopolymers are well-known for their superior resistance against chemical attack; hence, the disadvantage of their higher porosity is mitigated.

## 5. Conclusions

This study investigated the self-sensing properties of three different aluminosilicate composites, based on Portland cement, alkali-activated slag, and slag/fly ash geopolymer, with graphite powder as a conductive admixture. The main focus was on the comparison of the sensing sensitivity based on the measurement of changes in the electrical resistance and capacitance under compressive loading. The following conclusions can be drawn:

- Alkali-activated composites (AAS G6 and FAS G6) show better electrical response to applied compressive load than cement-based composite. On the one hand, this is caused by a lower initial electrical resistance, and on the other hand, it is associated with a less stiff structure and higher compressive strain.
- All the composites showed an increase in baseline resistance and a decrease in baseline capacitance during cyclic loading, which can be attributed to permanent changes in the microstructure. Alkali-activated composites appeared more prone to permanent microstructural damage even at low compressive stress, likely due to the closing of pre-existing microcracks. The analysis of permanent changes in the electrical properties can be used to detect microstructural changes that are not reflected in other parameters, for example, compressive strain.
- The values of the gauge factors showed that the resistance-based self-sensing ability was generally better than that of capacitance-based sensing for all the composites tested. The fly ash/slag geopolymer composite (FAS G6) demonstrated the highest sensitivity for both resistance-based and capacitance-based sensing, with gauge factor values approximately twice as high as those of the other composites, which was attributed to its higher ionic conductivity from soluble alkalis.
- Overall, the study demonstrated that alkali-activated composites with graphite filler have promising self-sensing capabilities based on both resistance and capacitance measurements, which could be advantageous for smart structure applications, despite some compromise in the mechanical properties. The cement-based composite showed very poor sensitivity for capacitance-based sensing; in this regard, capacitance-based sensing is not suitable for ordinary Portland cement concrete when a sandwich configuration of electrodes is applied. The considerable variability in the initial capacitance among different materials requires further investigation of the conditions (temperature and moisture) that have a significant effect on the electrical parameters and the assessment of their influence on the self-sensing sensitivity.

## Figures and Tables

**Table 1:** The chemical composition of aluminosilicate precursor analyzed by XRF method.

Composition	SiO_2_	Al_2_O_3_	Fe_2_O_3_	CaO	MgO	MnO	TiO_2_	SO_3_	Others
Slag (%)	41.64	7.81	0.35	36.25	8.06	0.59	0.46	1.21	3.63
Fly ash (%)	51.67	23.31	7.08	4.45	0.36	1.14	1.00	0.01	10.98
Cement (%)	20.98	5.47	3.84	59.67	1.31	0.10	0.26	2.51	5.86

**Table 2:** The mixing proportions.

Mixture	AAS G6	FAS G6	CEM G6
Slag (g)	1000	500	-
Fly ash (g)	-	500	-
Cement (g)	-	-	1000
Britesil (g)	200	400	-
Sand (g)	3000	3000	3000
Graphite (g)	60	60	60
Triton X-100 (mL)	30	30	30
Lukosan S (mL)	22	22	22
Water (mL)	415	404	400

**Table 3:** Initial electrical parameters and calculated gauge factors.

Mixture	CEM G6	AAS G6	FAS G6
Initial resistance (Ω)	9.81 × 10^5^	9.60 × 10^4^	180
*GF_R_*	45.0 ± 0.4	41.5 ± 0.7	86.1 ± 1.3
Initial capacitance (nF)	0.13	0.74	218
*GF_c_*	10.2 ± 0.3	24.0 ± 0.9	74.5 ± 1.0

## Data Availability

The original contributions presented in the study are included in the article, further inquiries can be directed to the corresponding author.
